# Risk factors for diabetes in recently arrived migrants in Scania, Sweden compared to the general population

**DOI:** 10.3389/fpubh.2025.1451669

**Published:** 2025-03-05

**Authors:** Slobodan Zdravkovic, Mathias Grahn, Elisabeth Mangrio, Margareta Rämgård, Magdalena Annersten Gershater

**Affiliations:** ^1^Department of Care Science, Faculty of Health and Society Malmö University, Malmö, Sweden; ^2^Malmö Institute for Studies of Migration, Diversity and Welfare Malmö University, Malmö, Sweden; ^3^Unit for Statistics and Analysis, Municipality of Malmö, Malmö, Sweden

**Keywords:** diabetes mellitus, education level, hypertension, migration, obesity, physical activity, risk factors, smoking

## Abstract

**Introduction:**

The prevalence of type 2 diabetes has increased worldwide, where the highest prevalence has been found in the Eastern Mediterranean region. Effective measures must be taken to prevent or delay the occurrence of type 2 diabetes and its complications. The present study aimed to investigate the correlation between factors linked to risk for diabetes, individually and cumulatively, and established diabetes in recently arrived migrants from Iraq and Syria in Scania and compare it to the rest of the population.

**Method:**

A cross-sectional survey was used to compare data between a sample of recently arrived migrants and a sample from the rest of the population in Scania, Sweden.

**Results:**

The prevalence of self-reported diabetes was significantly higher among recently arrived migrants (6.9%) compared to the rest of the population (4.9%). High blood-pressure, unhealthy weight, physical inactivity, and older age increased the risk for self-reported diabetes solely but also cumulatively.

**Conclusion:**

It is important to identify individuals with a high risk of diabetes and put preventive efforts into combating risk factors for diabetes. Targeting specific risk factors significantly reduces the risk of developing this disease.

## 1 Introduction

There has been an increase in diabetes prevalence worldwide, and in some regions, it has even doubled in the past few decades (1). In 2019, the overall world prevalence of diabetes was estimated at 8.3%, with around 10% diagnosed as type 1 and 90% as type 2 diabetes. The highest prevalence has been observed in the Eastern Mediterranean region, estimated at 12.2%, while in Europe it has been estimated to be 6.3% (2). Long standing diabetes is the cause of diabetes complications, including microvascular complications (such as nephropathy leading to ESRD, retinopathy leading to impaired vision or blindness, neuropathy leading to several symptoms including diabetic foot ulcers and amputation) (3) and macrovascular complications (such as cardiovascular disease, stroke, and peripheral vascular disease) (4, 5). This condition undoubtedly leads to a decreased quality of life for many patients (6).

The observed negative trend in diabetes incidence parallels the observed increase in the prevalence of obesity, which is considered as a key risk factor for type 2 diabetes as it contributes to insulin resistance, especially in combination with heredity and a sedentary lifestyle (2, 7). In addition, smoking increases insulin resistance and affects cardiovascular disease (8), and hypertension contributes to both cardiovascular complications and death (9). Several attempts have been made over the years to predict diabetes occurrence by identifying significant risk factors that can be measured through a risk score model (10, 11). Studies performed in Finland suggested that the factors age, heredity, physical activity, hypertension, waist circumstance, and body mass index (BMI) as well as high glucose intake and low daily consumption of fruit and vegetables constitute variables in the risk score (12). In Denmark, a similar approach measured the risk score based on the factors physical activity, hypertension, and BMI (13).

In recent years, Sweden has received a substantial number of immigrants from countries with a higher prevalence of type 2 diabetes (2) and higher prevalence of diabetes risk factors than the population already residing in Sweden (14). Overall diabetes prevalence in Sweden is 5% (2), and Scania has been average in the consumption of diabetes-lowering agents compared to other regions which indicates a prevalence in line with the rest of the country. Recently published reports have suggested that diabetes prevalence among recently arrived migrants in Scania is 6.2% and that the occurrence of diabetes, hypertension, overweight, and obesity is more frequent among migrants who have been in the region for 5 years or longer compared to those recently arrived (15).

As health care expenditures are increasing in the region, it is important to identify relevant risk factors among its population in order to undertake actions for preventing or delaying type 2 diabetes and its complications. Therefore, the primary aim of this study was to explore the correlation between known single risk factors for type 2 diabetes (age, BMI, hypertension, smoking, physical inactivity, and educational level) and established diabetes for recently arrived migrants in Scania compared to that correlation for a random sample of the Scania population. The secondary aim was to determine the cumulative effect of these risk factors on diabetes occurrence in both cohorts.

## 2 Methods

### 2.1 Study design

A regional survey of Scania based on a cross-sectional design was used to collect data on two cohorts established through random sampling. The first cohort consisted of 10,000 adults recently arrived migrants (RAM) born in Iraq or Syria, living in Scania, and having a residence permit approved between September 1, 2012, and August 31, 2016. The second cohort, which served as a reference comparison group (RCG), consisted of 10,000 individuals from the Scania population living in Sweden prior and up to August 31, 2012. Thus, a total of 20,000 individuals were invited to participate in the study. Both cohorts received a self-administrated health questionnaire covering various health and health-related questions (such as self-rated health, numerous diseases, drug and tobacco use, oral health, and mental health) and various lifestyle factors (such as physical activity, dietary habits, perceived stress, and sleeping patterns) as well as living conditions and employment. The questionnaire was initially developed in Swedish and then translated into Arabic for the Arabic speaking cohort by qualified translators. It was translated back and forth and reviewed for internal consistency by external Arabic/Swedish speaking reviewers. Data collection was performed through both paper-and web-based questionnaires between October and December 2018. The overall response rate was *n* = 6,566 (32.8%), including 3,461 men and 3,105 women with an overall mean age of 44.7 years (range 22–70 years).

### 2.2 Variables

The variables selected for this analysis were age, gender, BMI, hypertension, physical inactivity, smoking, level of education, and diabetes.

All variables were self-reported.

- Age and physical activity were treated as categorical variables.
- BMI was calculated from self-reported length and weight.
- Overweight was defined as BMI 25–30 kg/m^2^.
- Obesity was defined as BMI > 30 kg/m^2^.
- Hypertension was self-reported with either “yes” or “no” responses.
- Physical inactivity was estimated from the respondents’ responses about the amount of time per week spent being physically active during leisure time in terms of walking, bicycling, running, and playing sports.
- Smoking was self-reported without specifying type or amount.
- Level of education was divided into low (elementary school), middle (secondary school), and higher education (university).
- Diabetes was self-reported with either “yes” or “no,” without specifying its type.

### 2.3 Cumulative risk factor analysis

Cumulative risk was calculated by adding the grades of risk factors derived from the variables as follows: dichotomous variables were graded as 1 if present and as 0 if not. The variables age > 65 years, obesity (BMI > 30 kg/m^2^), hypertension, and physical inactivity were graded as 1 each, where a total of 4 indicates exposure to all risk factors, 3 indicates the presence of three factors, and so on.

### 2.4 Statistical analysis

The descriptive data were analyzed by frequencies and percentages. A chi-square test was used to test for differences in risk factor profiles between the two cohorts. Binary logistic regression was used to estimate the association between the outcome variable and the selected risk factors, both specific and cumulative, in terms of crude and adjusted odds ratios (OR), including 95% confidence intervals. The statistical software used was IBM SPSS version 22.

## 3 Results

The total response rate was 32% in the RAM cohort (*n* = 3,226), whereof 2,020 identified as men and 1,206 as women, and it was 33% in the RCG cohort (*n* = 3,340), whereof 1,441 were men and 1,899 were women. Regarding age, the RAM cohort was younger than the RCG cohort: RAM as compared to the RCG in all age categories (*p*-value <0.05) except the age category 65–80, where the difference was borderline significant (*p*-value = 0.056). Furthermore, low and medium education levels were more common in the RAM cohort compared to the RCG one, while a higher education level was more common in the latter <0.001 (see Table 1).

**Table 1:** Distribution of age, BMI, hypertension, smoking, physical activity and educational level.

Variable	All *n* (%)	RAM *n* (%)	RCG *n* (%)	*p*-value
Age (years)				< 0.001
18–34	1,700 (25.9)	1,095 (33.9)	605 (18.1)	
35–44	1,668 (25.4)	1,041 (32.3)	627 (18.8)	
45–54	1,507 (23.0)	742 (23.0)	765 (22.9)	
55–64	1,181 (18.0)	307 (9.5)	874 (26.2)	
65 +	510 (7.7)	41 (1.3)	469 (14.0)	
Total	6,566	3,226	3,340	
BMI				< 0.001
< 25 kg/m^2^	2,510 (39.7)	955 (31.0)	1,555 (47.8)	
25–30 kg/m^2^	2,462 (38.9)	1,328 (43.2)	1,134 (34.8)	
> 30 kg/m^2^	1,356 (21.4)	793 (25.8)	563 (17.3)	
Total	6,328	3,076	3,252	
Hypertension				< 0.001
Yes	891 (15.2)	338 (12.3)	553 (17.8)	
No	4,972 (84.8)	2,415 (87.7)	2,557 (82.2)	
Total	5,863	2,753	3,110	
Smoking				< 0.001
Yes	1,112 (17.3)	860 (27.4)	252 (7.6)	
No	5,330 (82.7)	2,274 (72.6)	3,056 (92.4)	
Total	6,442	3,134	3,308	
Physical inactivity				< 0.001
Active	3,373 (51.6)	1,005 (31.3)	2,368 (71.3)	
Inactive	3,164 (48.4)	2,208 (68.7)	956 (28.7)	
Total	6,537	3,213	3,324	
Educational level				< 0.001
Low	1,301 (19.9)	1,033 (32.4)	268 (8.1)	
Medium	1,805 (27.7)	720 (22.6)	1,085 (32.6)	
High	3,417 (52.4)	1,438 (45.1)	1,979 (59.4)	
Total	6,523	3,191	3,332	

*The results are presented as frequencies and percentages. *p*-values were calculated using the chi-square test.

Overweight (BMI 25–30 kg/m^2^) and obesity (BMI > 30 kg/m^2^) were common in both cohorts. In the whole RAM cohort, overweight was *n* = 1,328 (41.2%) and obesity was *n* = 793 (24.6%); in the whole RCG cohort, overweight was *n* = 1,134 (33.4%) and obesity was *n* = 563 (16.9%). Meanwhile, overweight in the RAM cohort without diabetes was *n* = 1,028 (42.3%) and obesity was *n* = 530 (21.8%), while overweight in the RCG cohort without diabetes was *n* = 970 (34.6%) and obesity was *n* = 450 (16.1%) (*p* = < 0.01). Men in the RAM cohort were more overweight (*n* = 699, 45.6%) or obese (*n* = 324, 21.1%) than those in the RCG who were overweight (*n* = 595, 42.6%) or obese (*n* = 230, 16.5%). In addition, women in the RAM cohort were more overweight (*n* = 329, 36.7%) than women in the RCG (*n* = 375, 26.7%) and more obese (*n* = 206, 23.0%) than those in the RCG (*n* = 220, 15.7%).

Self-reported hypertension was twice as common among persons in the RCG cohort (*n* = 287, 10.0%) than in the RAM cohort (*n* = 115, 4.5%) with the same distribution among men and women.

Smoking was more than three times as common among persons in the RAM cohort, where n = 860 (26.6%) of the respondents with diabetes stated that they smoke, compared to the RCG cohort, where *n* = 252 (7.5%) of the respondents with diabetes reported smoking.

Physical *inactivity* was more than twice as common in the RAM cohort (*n* = 1,681, 66.1%) than in the RCG cohort (*n* = 870, 30.4%). Women RCGs were more than three times more likely to be physically *active* (*n* = 999, 69.2%) than women in the RAM cohort (*n* = 200, 21.1%).

Regarding the education level, *n* = 1,033 (32%) of the RAM cohort reported elementary school as their highest level of education, compared to *n* = 242 (8%) of the RCG cohort. More persons in the RCG cohort reported their highest level of education as secondary school (*n* = 1,085, 32.5%) or higher education (*n* = 1,979, 59.3%) than persons in the RAM cohort, where *n* = 720 (22.3%) reported secondary school and *n* = 1,438 (44.6%) reported higher education (see Table 2).

**Table 2:** Distribution of age, BMI, hypertension, smoking, physical inactivity and educational level stratified by cohort and gender.

Variable	RAM male *n* (%)	RAM female *n* (%)	RCG male *n* (%)	RCG female *n* (%)	*p*-value
Age (years)					< 0.001
18–34	668 (33.1)	427 (35.4)	246 (17.1)	359 (18.9)	
35–44	642 (31.8)	399 (33.1)	251 (17.4)	376 (19.8)	
45–54	498 (24.6)	244 (20.2)	333 (23.1)	432 (22.7)	
55–64	192 (9.5)	115 (9.5)	379 (26.3)	495 (26.1)	
65 +	20 (1,0)	21 (1.7)	232 (16.1)	237 (12.5)	
Total	2,020	1,206	1,441	1,899	
BMI					< 0.001
< 25 kg/m^2^	562 (29.0)	393 (34.4)	523 (37.1)	1,032 (56.0)	
25–30 kg/m^2^	890 (46.0)	438 (38.4)	617 (43.8)	517 (28.1)	
> 30 kg/m^2^	483 (25.0)	310 (27.2)	270 (19.1)	293 (15.9)	
Total	1,935	1,141	1,410	1,842	
Hypertension					< 0.001
Yes	209 (11.9)	129 (12.9)	293 (21.6)	260 (14.8)	
No	1,544 (88.1)	871 (87.1)	1,062 (78.4)	1,495 (85.2)	
Total	1,753	1,000	1,355	1,755	
Smoking					< 0.001
Yes	704 (36.0)	156 (13.2)	80 (5.6)	172 (9.1)	
No	1,252 (64.0)	1,022 (86.8)	1,345 (94.4)	1,711 (90.9)	
Total	1,956	1,178	1,425	1,883	
Physical inactivity					< 0.001
Active	764 (38.0)	241 (20.1)	1,012 (70.7)	1,356 (71.7)	
Inactive	1,248 (62.0)	960 (79.9)	420 (29.3)	536 (28.3)	
Total	2,012	1,201	1,432	1,892	
Educational level					< 0.001
Low	639 (32.0)	394 (33.1)	134 (9.3)	134 (7.1)	
Medium	432 (21.6)	288 (24.2)	554 (38.5)	531 (28.1)	
High	928 (46.4)	510 (42.8)	752 (52.2)	1,227 (64.9)	
Total	1,999	1,192	1,440	1,892	

Self-reported diabetes was significantly more prevalent among the RAM cohort (*n* = 170, 6.2%) compared to RCG cohort (*n* = 147, 4.9%) (*p*-value = 0.02). In the RAM cohort, no difference was observed between men and women in relation to self-reported diabetes (*p*-value = 0.96); meanwhile, in the RCG cohort, gender was a significant factor (*p*-value = 0.015), with men having 1.5 times (95% CI 1.1–2.1) higher odds of self-reported diabetes compared to women (see Table 3).

**Table 3:** Self-reported prevalence of diabetes stratified by cohort and gender.

Variable	RAM male *n* (%)	RAM female *n* (%)	RCG male *n* (%)	RCG female *n* (%)
18–34	9 (8.4)	8 (12.7)	1 (1.1)	3 (5.1)
35–44	23 (21.5)	18 (28.6)	7 (8)	3 (5.1)
45–54	46 (43)	14 (22.2)	23 (26.1)	13 (22)
55–64	26 (24.3)	19 (30.2)	34 (38.6)	26 (44.1)
65–80	3 (2.8)	4 (6.3)	23 (26.1)	14 (23.7)
Total	107 (6.3)	63 (6.2)	88 (5.8)	59 (3.9)

The results are presented as frequencies and percentages.

### 3.1 Specific risk factor analyses

In the RAM cohort the estimated crude OR for overweight was significant in both RAM men (3.9, 95% CI 2.3–6.7) and women (7.9, 95% CI 3.5–17.5) as well as in RCG men (11.4, 95% CI 5.8–22.2) and women (7.6, 95% CI 3.7–15.6). The crude OR for hypertension was significant in both cohorts independently of gender (highest OR for RAM women) as well as for low level of education (highest OR for RCG women). Physical inactivity increases significantly the risk for self-reported diabetes in both RCG men and women as well as in RAM men but not in RAM women. The OR for smoking was significant in RCG women (2.3, 95% CI 1.2–4.4) (see Table 4).

**Table 4:** Crude OR and 95% confidence intervals stratified by cohort and gender.

Risk factor	RAM male OR (95% CI)	RAM female OR (95% CI)	RCG male OR (95% CI)	RCG female OR (95% CI)
BMI				
< 25 kg/m^2^	1	1	1	1
25–30 kg/m^2^	1.3 (0.7–2.2)	2.7 (1.1–6.3)	2.2 (1.1–4.4)	4.5 (2.2–9.3)
> 30 kg/m^2^	3.9 (2.3–6.7)	7.9 (3.5–17.5)	11.4 (5.8–22.2)	7.6 (3.7–15.6)
Hypertension				
No	1	1	1	1
Yes	47.6 (27.8–76.9)	50.0 (25.6–100.0)	22.2 (13.3–37.0)	22.7 (12.0–43.5)
Smoking				
No	1	1	1	1
Yes	1.0 (0.6–1.4)	1.5 (0.7–3.0)	0.9 (0.4–2.3)	2.3 (1.2–4.4)
Physical activity				
Active	1	1	1	1
Inactive	2.6 (1.6–4.2)	0.9 (0.5–1.8)	2.1 (1.4–3.3)	3.2 (1.9–5.4)
Educational level				
High	1	1	1	1
Medium	1.6 (0.9–2.6)	1.6 (0.7–3.7)	2.5 (1.5–4.0)	1.5 (0.8–2.8)
Low	1.7 (1.1–2.7)	4.1 (2.1–8.0)	3.0 (1.5–6.0)	4.4 (2.2–8.7)

### 3.2 Cumulative risk factor analyses

The results showed significant associations between all risk factors combined and self-reported established diabetes in both cohorts combined for men and for women, adjusted for level of education and cohort. The highest adjusted OR for men was observed in the risk score 3 (OR 102.8, 95% CI 50.1–211.3) and for women in the risk score 4 (OR 30.7, 95% CI 8.7–108.7). The more risk factors were present, the higher the OR was observed for women (see Table 5).

**Table 5:** Risk score OR and 95% confidence intervals by gender.

Variable	Men		Women	
	OR	95% CI	OR	95% CI
Risk score				
0	1		1	
1	6.5 ^a^	3.3–12.7	1.3	0.7–2.4
2	18.6 ^a^	9.5–36.6	5.8 ^a^	3.3–10.5
3	102.8 ^a^	50.1–211.3	27.7 ^a^	14.2–53.8
4	46.4 ^a^	10.6–203.0	30.7 ^a^	8.7–108.7

*Adjusted for level of education and cohort. ^a^*p* < 0.05.

Analyzing the risk factors based on the type of cohort and adjusting for gender and level of education resulted in significant OR for all risk scores in both the RAM and the RCG cohort (see Table 6).

**Table 6:** Risk score OR and 95% confidence intervals stratified by cohort.

Variable	RAM		RCG	
	OR*	95% CI	OR*	95% CI
Risk score				
0	1		1	
1	2.2^a^	1.2–4.0	4.7 ^a^	2.6–8.4
2	8.3 ^a^	4.5–15.2	13.1 ^a^	7.2–24.0
3	70.9 ^a^	35.1–143.0	48.0 ^a^	25.0–92.2
4	38.5 ^a^	5.8–254.2	46.3 ^a^	15.1–141.3

*Adjusted for gender and level of education. ^a^*p* < 0.05.

## 4 Discussion

### 4.1 Future risk of developing diabetes

Being a recent migrant did not constitute a risk for established diabetes, but the findings confirm that the combination of age, high BMI, hypertension, and physical inactivity increases the risk for developing diabetes. Independently of other diabetes-promoting factors such as heredity, these four factors are known to increase the risk of diabetes (11). In line with previous studies, this study shows that these four factors increased the risk of diabetes in both the RAM and the RCG cohorts. Therefore, they need to be taken into serious consideration by health care organizations. If no action is taken to reduce smoking, hypertension, overweight, and obesity, there will be an increase in demands for adequate diabetes care in primary care as well as in hospitals and municipal home care within the near future.

The high self-reported prevalence in the 18–54 years age group is worrying as diabetes is a chronic condition; they will get older with diabetes, so health care organizations targeting older people need to prepare for more patients with diabetes and complications in the near future.

Overweight and obesity were common in both cohorts in both men and women. This is in line with the growth of obesity in Swedish society and in Europe in general (7). Obesity is a complex and heterogeneous chronic disease. It also predisposes the individual to the development of other medical complications than type 2 diabetes, such as nonalcoholic fatty liver disease and some common cancers (16). It requires individualized treatment and long-term support and is best treated with evidence-based methods such as adequate nutrition and exercise, psychological and behavioral interventions, pharmacotherapy, and bariatric surgery (17). As this disease is chronic in nature, the treatment plan must be personalized, long term, and designed in agreement with the patient, addressing the drivers of weight gain.

Physical inactivity was more common in the RAM cohort than in the RCG cohort in both men and women. This may reflect difficulties in integration (18); namely, it takes migrants several years to become established in the Swedish society, and unemployed persons tend to be less physically active. Moreover, modern apartments do not contribute to a physically active lifestyle.

Hypertension was reported as more common among the RCG than the RAM cohort in both men and women. The reason for this is unclear; however under-diagnosis may play a role here as well as it might also be due to the differences in age distribution between the two cohorts where the respondents in the RAM were younger which is in line with a previous study regarding such association (19). Optimal treatment of hypertension is of importance, especially in persons with type 2 diabetes, in order to reduce morbidity and mortality (20).

Smoking was twice as common among persons in the RAM cohort as the RCG cohort. Persons who reporting having diabetes also reported being active smokers. This behavior may be attributed to smoking being a socially accepted drug to self-treat the stress that comes with moving to a new country but not being integrated into the society. This group of smokers with diabetes urgently needs interventions to promote smoking cessation. Studies have shown that individually-delivered smoking cessation counseling can assist smokers to quit (21). Providing such interventions for patients with diabetes might be a challenge for primary care.

Regarding the level of education, more persons in the RCG cohort had secondary school or higher education than persons in the RAM cohort, which could be attributed to differences in educational systems between Sweden and Syria and Iraq. Similarly, a Swedish study by Wemrell et al. (22) found that type 2 diabetes risk was higher among immigrants and among individuals with low educational achievement, compared to persons born in Sweden and individuals with high educational achievement. The influence of education level on diabetes risk is related to the socioeconomic situation. That is, with a lower education level, recently arrived migrants risk a low socioeconomic situation with unemployment and segregation. McGavock et al. (23) pointed out that type 2 diabetes in young people is largely a disease of poverty and that young-onset type 2 diabetes is strongly associated with relative socioeconomic deprivation.

### 4.2 Diabetes prevalence

The present study used data from adults self-reporting on diabetes independently of its type. The self-reported diabetes prevalence in the RAM cohort (6.2%) is higher than the Swedish official prevalence (5%), which is more in line with prevalence in the RCG cohort (4.9%). Diabetes prevalence was higher among men (6.7%) than among women (4.7%). Moreover, the risk increased with age to the extent that the prevalence was more than seven times higher in the oldest age group than in the youngest. These findings should mainly be interpreted as referring to type 2 diabetes since this type of diabetes is most common in adults (2). Furthermore, as diabetes in this study is self-reported, the observed prevalence only covers those being aware of having the diagnosis, which means that it might be underestimated. The prevalence of diabetes in Sweden in 2013 was 6.8% (24), and the worldwide prevalence was 10.5% (2). The results of the present study are below these figures.

The factors addressed in the present study are well-known risk factors for the development of diabetes, whereof some are modifiable. Furthermore, the risk of developing diabetes increases as exposure to more than one risk factor occurs (12). Several prediction models for the development of diabetes have been suggested over the years, including both invasive and non-invasive measures (25, 26). For example, Finrisk is a model originally applied to a Finnish population but has since been widely used and tested in different populations (27). The validity of the prediction models has also been tested but with varying results in terms of short or long prediction of diabetes occurrence (28). Predicting diabetes might prevent the disease from occurring, and delay the disease deterioration, or delay the occurrence of diabetes complications (26).

Unhealthy weight and sedentary lifestyle are two important factors in the work of diabetes prevention. The recently arrived migrants included in the present study are Arabic speaking, migrating from Syria or Iraq. Previous studies in Sweden have shown that this group is physically inactive, smoking (men), mainly unemployed, and at a moderate risk of mental illness (29). Having a migrant background increases the risk of several diseases (15). The healthy migrant effect (30) suggests that the health of migrants seems to decline after a number of years in the new country. A recently published report from the Swedish Public Health Agency suggests that diabetes, high blood pressure, and obesity and overweight are more prevalent in migrants living in Sweden for 5 years as compared to those recently arrived (15).

The Rule of Halves applied on data from Copenhagen suggested that the percentage of those having undiagnosed diabetes was 26% (13). However, as the present study compares recently arrived migrants with the reference population of Scania, it is important to understand the proportion of undiagnosed diabetes cases in both groups. The estimate of undiagnosed diabetes in Sweden when screening for diabetes type 2 was 54% (31). Self-reported diabetes is a strength to those who are aware of their conditions; it provides a good foundation for active self-care and adherence to a healthy lifestyle. In Sweden, health checks are offered to asylum seekers in their initial phase in the country, but the percentage of those attending the health checks is rather low, approximately 34% (32). As the present study confirms, diabetes prevention programs should target lifestyle behaviors as they constitute risk factors. Health-promoting urban initiatives—such as the diabetes prevention initiative Cities Changing Diabetes and the community-based, participatory, and challenge-driven program aiming at improving health in an ethnically diverse low-income neighborhood of Malmö, Sweden (33) are important, not least in vulnerable groups. Cities Changing Diabetes focuses on mapping the prevalence of diabetes and its risk factors, sharing the knowledge to various stakeholders, and taking action to prevent its occurrence in various cities of the world (34).

Our findings suggest that diabetes prevalence in migrants is larger than that observed in the reference population as well as in previous public health surveys (15, 35). It is of uttermost importance to systematically identify individuals at high risk and apply an individualized primary prevention strategy in the population to ensure the best health outcome.

## 5 Strengths and limitations

The response rate for the present study was 32.8%, which could be considered fairly high in regard to studies including migrants, even if it is somewhat lower than other similar surveys (34, 36). The data used in this study is self-reported, which reduces its reliability. For example, some of the respondents may not have been aware of health issues such as blood pressure or diabetes because they had not recently been to a medical appointment. A risk factor such as smoking might be underreported since it is well-known that smoking affects health negatively. Accordingly, the reported number of smokers should be considered as the minimum of the true prevalence. Another limitation is that the questions in the survey are of a sensitive nature, which could increase the chances of respondents not being completely truthful. The strength of the study is that it covers an entire region, and the use of an Arabic translation ensured that the survey reached all recently arrived migrants, who often do not respond to similar surveys due to language issues. In addition, the survey was translated back and forth by authorized translators, which increases the chance of the questions being well understood by the migrants.

## 6 Conclusion

This study confirms the impact that the key risk factors have on diabetes occurrence in the population of Scania. High blood pressure, overweight, physical inactivity, and old age increase the risk of diabetes independently but also cumulatively; that is, being exposed to several factors markedly increases the risk. Lifestyle-related factors such as unhealthy weight and physical inactivity are more prevalent in recently arrived migrants. The present study confirms the impact of sedentary lifestyle on diabetes and highlights the importance of preventing the examined risk factors in the population of Scania, Sweden.

## Data Availability

The dataset presented in this article could be made available upon reasonable request. Requests to access the datasets should be directed to slobodan.zdravkovic@mau.se.
